# Increased sTREM-1 plasma concentrations are associated with poor clinical outcomes in patients with COVID-19

**DOI:** 10.1042/BSR20210940

**Published:** 2021-07-22

**Authors:** Aline H. de Nooijer, Inge Grondman, Simon Lambden, Emma J. Kooistra, Nico A.F. Janssen, Matthijs Kox, Peter Pickkers, Leo A.B. Joosten, Frank L. van de Veerdonk, Marc Derive, Sebastien Gibot, Mihai G. Netea, on behalf of RCI-COVID-19 study group

**Affiliations:** 1 Department of Internal Medicine, Radboud University Medical Center, 6500 HB Nijmegen, The Netherlands; 2 Radboudumc Center for Infectious Diseases, Radboud University Medical Center, 6500 HB Nijmegen, The Netherlands; 3 Department of Medicine, University of Cambridge, Cambridge, CB20 QQ, UK; 4 Department of Intensive Care Medicine, Radboud University Medical Center, 6500 HB Nijmegen, The Netherlands; 5 Núcleo de Pesquisa da Faculdade da Polícia Militar (FPM) do Estado de Goiás, Goiânia, Goiás, Brasil; 6 Inotrem, 54500 Vandoeuvre-les-Nancy, France; 7 Intensive Care Unit, Centre Hospitalier Regional Universitaire (CHRU), 54000 Nancy, France; 8 Immunology and Metabolism, Life & Medical Sciences Institute, University of Bonn, 53115 Bonn, Germany

**Keywords:** coronavirus disease 2019 (COVID-19), severe acute respiratory syndrome coronavirus 2 (SARS-CoV-2), acute respiratory distress syndrome (ARDS), inflammation, sTREM-1, mortality

## Abstract

Patients with sepsis display increased concentrations of sTREM-1 (soluble Triggering Receptor Expressed on Myeloid cells 1), and a phase II clinical trial focusing on TREM-1 modulation is ongoing. We investigated whether sTREM-1 circulating concentrations are associated with the outcome of patients with coronavirus disease 2019 (COVID-19) to assess the role of this pathway in COVID-19. This observational study was performed in two independent cohorts of patients with COVID-19. Plasma concentrations of sTREM-1 were assessed after ICU admission (pilot cohort) or after COVID-19 diagnosis (validation cohort). Routine laboratory and clinical parameters were collected from electronic patient files. Results showed sTREM-1 plasma concentrations were significantly elevated in patients with COVID-19 (161 [129–196] pg/ml) compared to healthy controls (104 [75–124] pg/ml; *P*<0.001). Patients with severe COVID-19 needing ICU admission displayed even higher sTREM-1 concentrations compared to less severely ill COVID-19 patients receiving clinical ward-based care (235 [176–319] pg/ml and 195 [139–283] pg/ml, respectively, *P* = 0.017). In addition, higher sTREM-1 plasma concentrations were observed in patients who did not survive the infection (326 [207–445] pg/ml) compared to survivors (199 [142–278] pg/ml, *P*<0.001). Survival analyses indicated that patients with higher sTREM-1 concentrations are at higher risk for death (hazard ratio = 3.3, 95%CI: 1.4–7.8). In conclusion, plasma sTREM-1 concentrations are elevated in patients with COVID-19, relate to disease severity, and discriminate between survivors and non-survivors. This suggests that the TREM-1 pathway is involved in the inflammatory reaction and the disease course of COVID-19, and therefore may be considered as a therapeutic target in severely ill patients with COVID-19.

## Introduction

The current coronavirus disease 2019 (COVID-19) pandemic has, to date, led to almost three million deaths (World Health Organization [WHO]) and caused massive societal and economic disruption. Severe acute respiratory syndrome coronavirus 2 (SARS-CoV-2), the pathogen responsible for COVID-19, can cause a variety of symptoms ranging from mild upper respiratory tract illness with no need for hospital admission to acute respiratory distress syndrome (ARDS) requiring admission to the hospital’s Intensive Care Unit (ICU) [1, 2]. To identify patients with an unfavourable disease course, previous research has focused on biomarkers for COVID-19 prognosis and showed a relationship between disease severity and elevated plasma concentrations of C-reactive protein (CRP), D-dimer, and several inflammatory cytokines, especially interleukin (IL)-6 [2–5]. However, it remains challenging to predict the prognosis early in the disease course of COVID-19, and more specific biomarkers are needed. In addition, elucidating the pathophysiological mechanisms could pave the way for identifying novel therapeutic targets and new host-directed therapies [6, 7].

One pathway extensively investigated in sepsis and other acute and chronic inflammatory conditions is Triggering Receptor Expressed on Myeloid cells 1 (TREM-1). TREM-1 is an immunoglobulin-like receptor expressed by innate immune cells that is known to amplify the innate immune response. Research has established that TREM-1 is up-regulated in bacterial, viral and fungal infections, with release of the cell surface portion of the TREM-1 receptor, soluble TREM-1 (sTREM-1) into the blood following activation of the receptor [8]. A meta-analysis of the studies published on the diagnostic and prognostic value of sTREM-1 in sepsis demonstrated a clear correlation between increased sTREM-1 plasma concentrations and mortality [9]. In a first publication on sTREM-1 in COVID-19, Van Singer et al. reported a relationship between increased sTREM-1 plasma concentrations, disease severity and clinical outcomes such as intubation and oxygen requirement [10].

In addition to the use of sTREM-1 as a prognostic biomarker, research has focused on modulation of the TREM-1 pathway. First, studies in mice show a favourable effect of transgenic or pharmacological TREM-1 modulation on sepsis survival [11, 12]. In a recent phase IIa study, patients with septic shock were treated with nangibotide, a specific TREM-1 modulator [13], demonstrating its safety and displaying trends towards clinically relevant benefits following administration. A phase IIb clinical trial in adults with septic shock is underway to explore this (NCT 04429334).

Whether the TREM-1 pathway has either diagnostic or therapeutic value in COVID-19 is not yet known. To assess this, research on the use of sTREM-1 as a predictive biomarker for selection of patients for this treatment is warranted. The present study aims to investigate the behaviour of sTREM-1 plasma concentrations in COVID-19 patients and its relation to disease severity and clinical outcomes. Furthermore, we assess whether patients with COVID-19 could be stratified based on sTREM-1 plasma concentrations and therefore could possibly benefit from anti-TREM-1-therapy.

## Materials and methods

### Study design and patients

Our retrospective study included two independent cohorts: a pilot cohort of 21 healthy volunteers and 24 patients with COVID-19 admitted to the ICU of Centre Hospitalier Régional Universitaire de Nancy, France, and a validation cohort of 192 patients with COVID-19 (both ICU and non-ICU) admitted to the Radboud University Medical Centre (Radboudumc), Nijmegen, the Netherlands.

The pilot cohort consists of 24 adult patients diagnosed with COVID-19 according to WHO interim guidance and admitted to the ICU between 15 March and 31 March 2020. All patients were positive for SARS-CoV-2 by polymerase chain reaction (PCR) and were admitted to the ICU for ARDS. The present study was approved by the local ethics committee (Saisine n°196).

The validation cohort consists of patients with a PCR-proven or clinically diagnosed SARS-CoV-2 infection admitted to the Radboudumc between 6 March and 15 April 2020. Clinical diagnosis of COVID-19 infection was defined based on signs and symptoms, specific computed tomography (CT) findings according the Dutch COVID-19 Reporting and Data System (CO-RADS) classification [14], and final consensus of clinical experts. 95% (183/192) of the COVID-19-diagnosed patients had a positive PCR at the time of diagnosis. The study protocol was approved by the local ethics committee (CMO 2020 6344 and CMO 2016 2963). All patients or legal representatives were informed about the study details and could decline to participate. Ethylenediaminetetraacetic acid (EDTA) plasma was collected at the first routine blood withdrawal for laboratory testing after COVID-19 diagnosis in the hospital. For analysis, patients were stratified into groups based on disease severity and mortality. We defined disease severity based on the need for ICU admission during hospital stay: severe illness in patients requiring ICU admission and moderate illness in patients for whom only ward based care was necessary. Ten patients were admitted to a non-ICU ward at time of sampling, but needed ICU admission later during their hospital stay. We have classified these patients in the severe illness group.

### Data collection

For the pilot cohort, medical records were collected and retrospectively analysed. Clinical data and laboratory results of the validation cohort were collected from electronic patient files (EPIC, EPIC Systems Corporation, Verona, WI, U.S.A.) and recorded in electronic Case Report Forms (Castor EDC, Amsterdam, the Netherlands). Values of white blood cell (WBC) counts, lymphocyte counts, and plasma concentrations of C-reactive protein (CRP), ferritin, and D-dimer were collected at the day of plasma sampling. Clinical outcomes (ICU admission, hospital length of stay [LOS], ICU LOS, incidence of thromboembolic events [TEE], and mortality) were recorded until hospital discharge.

### Measurements and assays

Venous blood was collected in EDTA tubes and subsequently centrifuged at 2954*** g*** (3800 RPM) at room temperature for 10 min. Plasma was collected and aliquoted before storage at −80°C for further analysis. Concentrations of IL-6 were measured using enzyme-linked immunosorbent assays (ELISA, Quantikine®, R&D systems) according to the manufacturer’s protocol, with a lower detection limit of 16 pg/ml. Plasma sTREM-1 concentrations were measured using an analytically validated ELISA assay according to regulatory requirements (EMA 2011) using a commercially available, research use only ELISA assay (Human TREM-1 Quantikine® ELISA kit, R&D Systems). This method was validated with lower and upper limits of quantification of 34.2 and 2070 pg/ml, respectively. For routine analysis, each analytical run contained three levels of quality control (QC) sample (low, mid and high), and each run is accepted if standard curve and QC samples are within acceptance criteria. The analytical performances and acceptance criteria of this method are summarized in Supplementary Table S1.

### Statistical analysis

The obtained data were analysed using SPSS version 25.0 (IBM Corp., Armonk, NY, U.S.A.), GraphPad Prism version 8.0 (GraphPad Software, Inc., San Diego, CA, U.S.A.), and MedCalc version 19.6.4 (MedCalc Software Ltd, Ostend, Belgium). Differences between groups were assessed by Mann–Whitney *U* tests for continuous variables and by Fisher’s exact tests for categorical variables. Correlations between sTREM-1 and inflammatory parameters were assessed by Spearman’s rank correlation tests. Linear regression analyses were used to assess the associations of sTREM-1 concentrations and symptom duration, hospital LOS, and ICU LOS. Receiver operating characteristic (ROC) analyses were performed to assess the prognostic performance of several biomarkers by calculating the area under the curve (AUC). The optimal cut-off values for the biomarkers was defined based on the maximal Youden’s J index and used to assess differences in survival during hospital admission for high versus low biomarker concentrations by Kaplan–Meier survival analysis. Hazard ratios were based on the log-rank (Mantel-Cox) test. A *P*-value <0.05 (two-tailed) was considered statistically significant.

## Results

### sTREM-1 circulating concentrations are higher in COVID-19 patients

**Figure 1:**
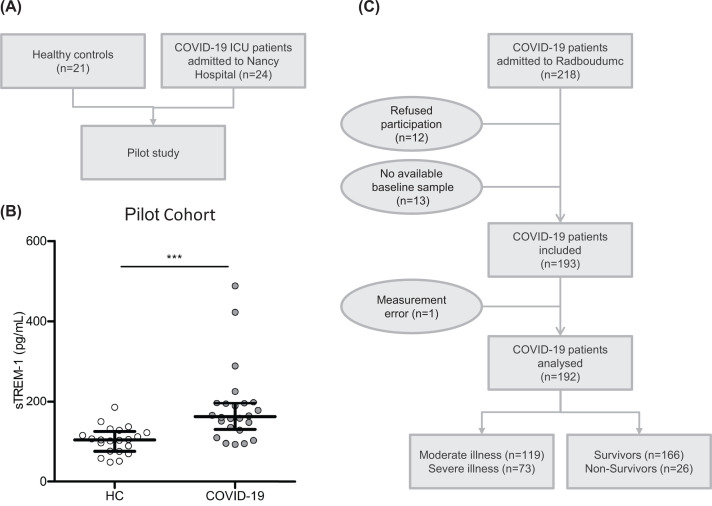
Study outline and results of the pilot cohort Pilot cohort (**A**) flow chart and (**B**) sTREM-1 plasma concentrations for HC versus COVID-19 patients. (**C**) Flow chart of validation cohort. Severe illness was defined as the need for ICU admission during hospital stay. Data are presented as median with interquartile range. *P*-value was calculated with Mann–Whitney *U* test. ***: *P*<0.001. Abbreviations: COVID-19, coronavirus disease 2019; sTREM-1, soluble Triggering Receptor Expressed on Myeloid cells 1; HC, healthy controls.

sTREM-1 concentrations were first measured in a cohort of 24 patients with COVID-19 admitted to the ICU of Nancy Hospital (Figure 1A). The characteristics of these patients are provided in Supplementary Table S2. This analysis demonstrated significantly higher sTREM-1 plasma concentrations for patients compared to 21 healthy controls (HC: 104 [75–124] pg/ml versus COVID-19: 161 [129–196] pg/ml, *P*<0.001, Figure 1B).

### Elevated sTREM-1 concentrations in patients with severe COVID-19

A total of 218 patients diagnosed with COVID-19 and admitted to the Radboudumc were assessed for study inclusion (Figure 1C). Of these, 12 patients refused to participate. Moreover, 13 other patients were excluded because no plasma sample was available from first routine blood sampling after COVID-19 diagnosis (baseline), and one patient was excluded due to a measurement error. The final study population (*n*=192) was divided in groups based on disease severity (moderate illness [*n*=119] and severe illness [*n*=73]) and outcome (survivors [*n*=166] and non-survivors [*n*=26]). Table 1 shows the characteristics of these patients, divided in groups of different disease severity and mortality. Severely ill patients had higher concentrations of inflammatory parameters (CRP, ferritin, and IL-6), a longer hospital LOS (31 days versus 7 days, *P*<0.001), and a higher mortality rate (22% versus 8%, *P* = 0.010) compared to the patients admitted to the clinical wards. Non-survivors were older than survivors (73 versus 64 years, *P*<0.001) and were more frequently admitted to the ICU (62% versus 34%, *P* = 0.010). No other relevant differences in gender, BMI, and comorbidities were observed between the groups.

**Table 1:** Patient characteristics validation cohort

	All patients (*n*=192)	Moderate illness (*n*=119)	Severe illness (*n*=73)	*P*-value^3^	Survivors (*n*=166)	Non-survivors (*n*=26)	*P*-value^4^
Age (years)	65 (54–72)	66 (53–73)	64 (57–71)	0.285	64 (53–71)	73 (69–75)	<0.001
Sex (*n*, %)							
Male	133 (69)	79 (66)	54 (74)	0.334	115 (69)	18 (69)	1.000
Female	59 (31)	40 (34)	19 (26)		51 (31)	8 (31)	
BMI (kg/m^2^)	26.4 (24.0–29.0)	26.0 (23.7–28.9)	26.8 (24.7–29.3)	0.250	26.6 (23.9–29.3)	25.8 (24.0–28.4)	0.651
Comorbidity (*n*, %)							
Diabetes mellitus	38 (20)	22 (19)	16 (22)	0.580	33 (20)	5 (19)	1.000
Cardiovascular disease	102 (53)	64 (54)	38 (52)	0.882	84 (51)	18 (69)	0.092
Pulmonary disease	39 (20)	31 (26)	8 (11)	**0.016**	31 (19)	8 (31)	0.188
Malignancy	48 (25)	34 (29)	14 (19)	0.171	41 (25)	7 (27)	0.810
Days of illness at day of sampling (days)	11 (8–15)	10 (8–14)	13 (9–16)	**0.022**	11 (8–15)	11 (8–17)	0.882
ICU admission (*n*, %)^1^	73 (38)	NA	NA	NA	57 (34)	16 (62)	**0.010**
Hospital length of stay (days)	9 (6–24)	7 (5–9)	31 (19–45)	**<0.001**	9 (6–24)	15 (6–15)	0.339
Mortality (*n*,%)	26 (14)	10 (8)	16 (22)	**0.010**	NA	NA	NA
sTREM-1 (pg/ml)	208 (151–292)	195 (139–283)	235 (176–319)	**0.017**	199 (142–278)	326 (207–445)	**<0.001**
CRP (mg/l)	109 (60–173)	81 (41–120)	175 (128–291)	**<0.001**	103 (56–172)	139 (83–210)	0.154
Ferritin (µg/l)	1058 (543–1879)	822 (399–1461)	1694 (935–2554)	**<0.001**	996 (491–1864)	1270 (717–1962)	0.237
IL-6 (pg/ml)^2^	72 (28–118)	43 (23–82)	144 (79–405)	**<0.001**	67 (26–104)	188 (70–480)	**<0.001**

Data are presented as median (IQR) or *n* (%). Abbreviations: BMI, body mass index; COVID-19, coronavirus disease 2019; CRP, C-reactive protein; ICU, intensive care unit; IL-6, interleukin-6; sTREM-1, soluble Triggering receptor Expressed on Myeloid cells 1.

1 ICU admission during total hospital admission.

2 measured in 151 of the 192 patients.

^3^moderate versus severe illness (Mann–Whitney *U* test).

4 survivors versus non-survivors (Mann–Whitney *U* test).

**Figure 2:**
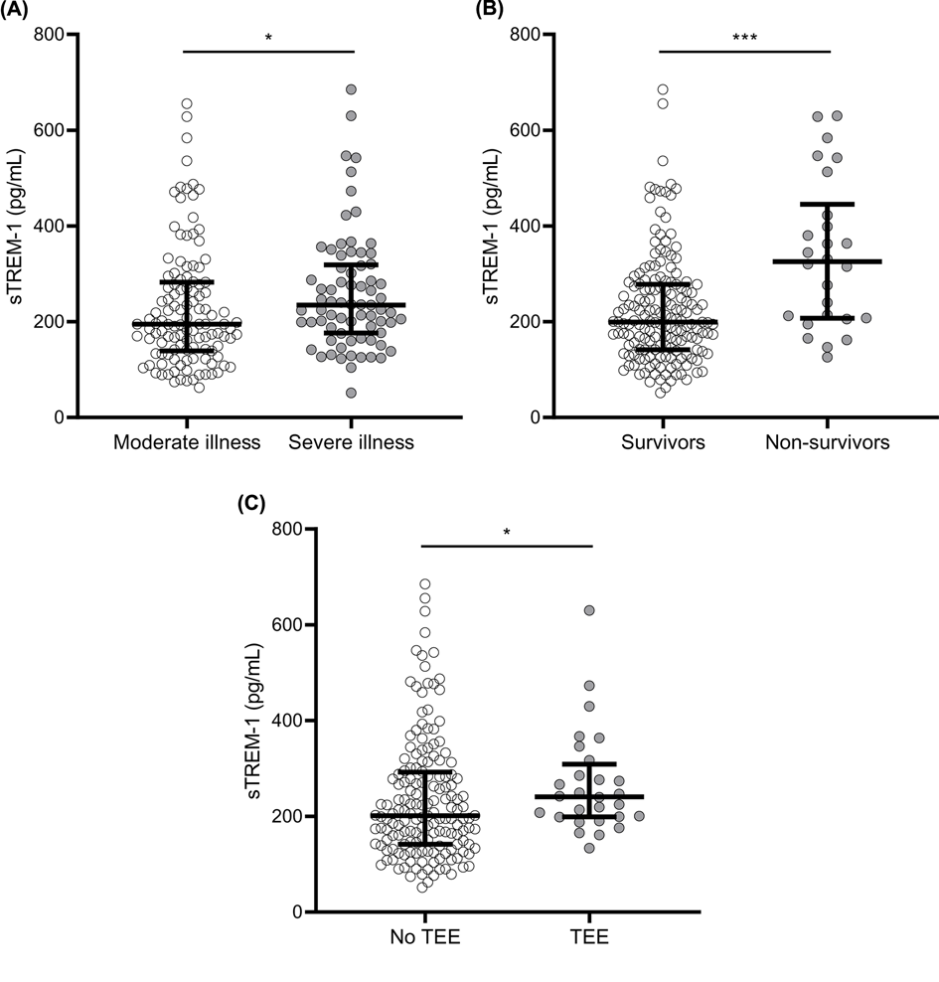
sTREM-1 plasma concentrations in different groups of patients with COVID-19 sTREM-1 plasma concentration for (**A**) moderate and severely ill patients, (**B**) survivors and non-survivors, and (**C**) patients with and without a TEE. Severe illness was defined as the need for ICU admission during hospital stay. Data are presented as median with interquartile ranges. Exact *P*-values are 0.017 (**A**), <0.001 (**B**), 0.044 (**C**). *P*-values were calculated with Mann–Whitney *U* tests. *: *P*<0.05, ***: *P*<0.001. Abbreviations: COVID-19, coronavirus disease 2019; ICU, intensive care unit; sTREM-1, soluble triggering receptor expressed on myeloid cells 1; TEE, thromboembolic event.

Figure 2 demonstrates higher sTREM-1 plasma concentrations in severely ill patients compared to patients with moderate illness (Figure 2A, 235 [176-319] pg/ml versus 195 [139–283] pg/ml, *P*=0.017). Similarly, non-survivors had higher sTREM-1 plasma concentrations compared to survivors (Figure 2B, 326 [207–445] pg/ml versus 199 [142–278] pg/ml, *P*<0.001). Furthermore, patients who developed thromboembolic events (*n*=28, 14%) presented with increased sTREM-1 concentrations compared to patients without this complication (Figure 2C, 241 [199–309] pg/ml versus 201 [142–292] pg/ml, *P*=0.044). Thromboembolic events encompassed pulmonary embolism (*n*=26), cerebrovascular accident (*n*=2), and deep venous thrombosis (*n*=1). The relationship between sTREM-1 and disease severity or mortality was separately assessed in these subgroups, indicating the largest difference in sTREM-1 concentrations between survivors versus non-survivors (Supplementary Figure S1). Additionally, sTREM-1 baseline concentrations were not associated with the total duration of hospital stay and ICU stay (Supplementary Figure S2BC, *R*^2^ = 0.019, *P*=0.057 and *R*^2^ = 0.006, *P*=0.506, respectively) and sTREM-1 concentrations were independent of symptom duration at time of sampling (*R*^2^ = 0.002, *P*=0.535, Supplementary Figure S2A). The latter finding suggests that sTREM-1 is persistently increased during the disease course in COVID-19 patients, but longitudinal measurements are warranted to prove this in future studies.

Significant correlations were observed for sTREM-1 concentration and WBC count, lymphocyte count, CRP, ferritin, D-dimer, and IL-6 (Supplementary Figure S3A–F). These findings indicate that sTREM-1 concentrations are directly associated with a systemic inflammatory response, although the increase in sTREM-1 concentrations is not fully determined by the classical inflammatory markers.

### Discriminatory power of sTREM-1 on mortality

**Figure 3:**
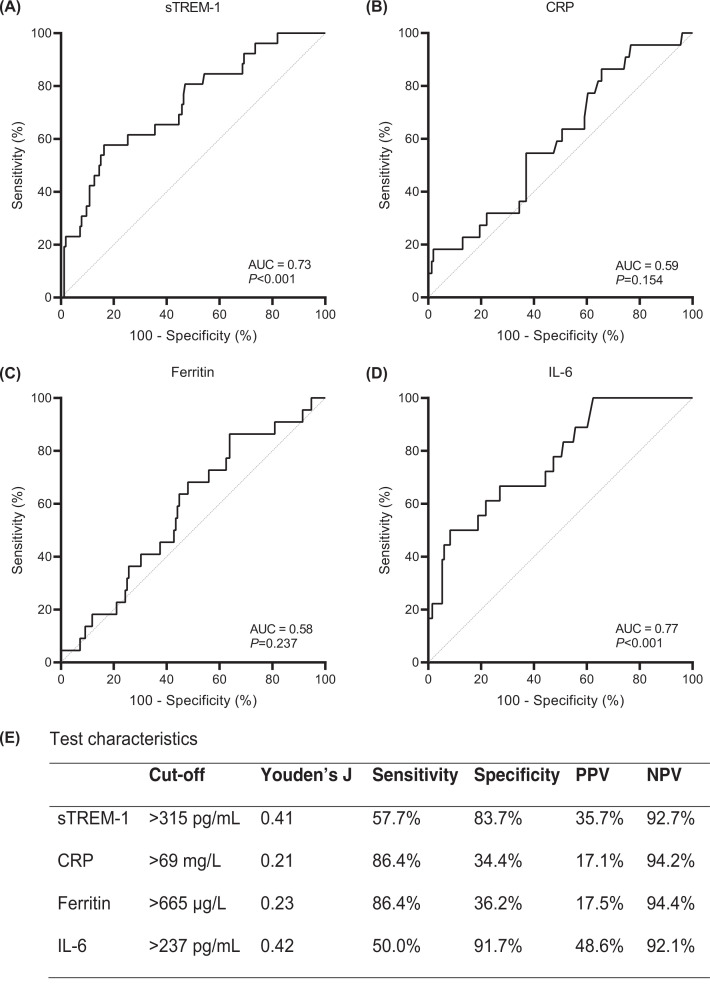
Discriminatory power for mortality in patients with COVID-19 Receiver-operating characteristic curves based on mortality for (**A**) sTREM-1, (**B**) CRP, (**C**) ferritin, and (**D**) IL-6 circulating concentrations and (**E**) the characteristics of these tests. Abbreviations: AUC, area under the curve; COVID-19, coronavirus disease 2019; CRP, C-reactive protein; IL-6, interleukin-6; sTREM-1, soluble Triggering Receptor Expressed on Myeloid cells 1; NPV, negative predictive value; PPV, positive predictive value.

**Figure 4:**
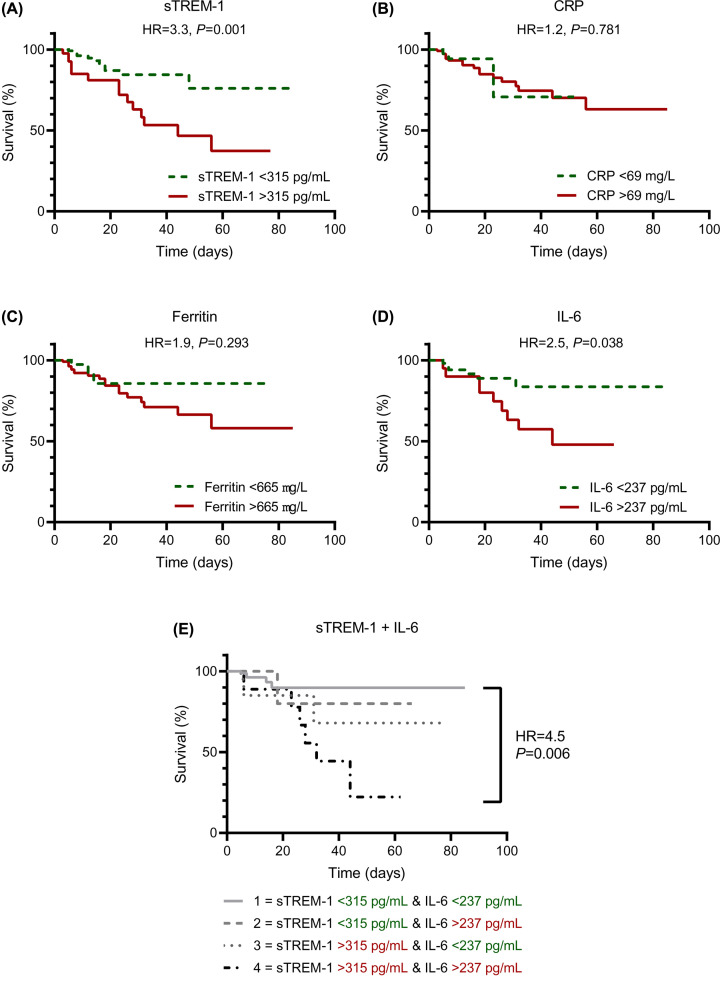
Survival analysis Percentage of survival of patients with COVID-19 for high versus low concentrations of (**A**) sTREM-1, (**B**) CRP, (**C**) ferritin, (**D**) IL-6, and (**E**) combination of sTREM-1 and IL-6. Cut-off values were based on the maximal Youden's J index derived from the receiver-operating characteristic curves (Figure 3). Hazard ratios were calculated with the log-rank (Mantel-Cox) test. Abbreviations: COVID-19, coronavirus disease 2019; CRP, C-reactive protein; HR, hazard ratio; IL-6, interleukin-6; sTREM-1, soluble Triggering Receptor Expressed on Myeloid cells 1.

Figure 3A presents the ROC-curve for discrimination between survivors and non-survivors based on sTREM-1 concentrations; the ROC-curves for CRP, ferritin and IL-6 are presented for comparison (Figure 3B–D). The test characteristics of the ROC analyses are presented in Figure 3E. The AUC for sTREM-1 was 0.73 (95%CI: 0.62–0.83) indicating moderate discrimination between survivors and non-survivors. This discriminatory power of sTREM-1 was similar to that of IL-6 (AUC = 0.77, 95%CI: 0.65–0.88; *P*=0.887), but performed better than CRP (AUC = 0.59, 95%CI: 0.47–0.72; *P*=0.132) and ferritin (AUC = 0.58, 95%CI: 0.46–0.70; *P*=0.078), although not significantly different. Next, patient survival analysis stratified for sTREM-1 indicates that patients with sTREM-1 plasma concentrations of more than 315 pg/ml had an increased risk for in-hospital mortality (hazard ratio = 3.3, 95%CI: 1.4–7.8, Figure 4A). Hazard ratios of mortality for high circulating concentrations of CRP, ferritin, and IL-6 were 1.2 (95%CI: 0.4–3.7), 1.9 (95%CI: 0.7–5.1), and 2.5 (95%CI: 0.9–6.9), respectively (Figure 4B–D). When combining the cut-off values for both sTREM-1 and IL-6, the hazard ratio was 4.5 (95%CI: 1.1–18.9, Figure 4E), implying a synergistic association between sTREM-1 and IL-6 in non-survivors.

## Discussion

The results of our observational study validate the hypothesis that sTREM-1 concentrations are increased in patients with COVID-19 and show that they are correlated with severity of the disease and subsequently with mortality. These findings suggest that the TREM-1 pathway may play a role in the disease course in COVID-19, and is a potential target for adjunctive immunotherapy.

Next to research into effective vaccines and potential antiviral drugs, numerous studies have focused on host-directed strategies as an adjunctive approach to improve the outcome of COVID-19 patients [6, 15], especially anti-inflammatory therapies such as anti-IL-6R antibodies or recombinant IL-1Ra. The beneficial impact of dexamethasone, acting most likely through its immune-modulating activities, on COVID-19 has been demonstrated [16], and early findings of the REMAP-CAP trial reported a beneficial effect of anti-IL-6R therapy in patients with COVID-19 admitted to the ICU [17]. However, not all patients respond favourably to these treatments and additional therapies are urgently needed. In the present study, we demonstrate that sTREM-1 circulating concentrations are elevated in patients with COVID-19, and they are strongly correlated with mortality.

It is interesting to note that, regarding the subgroup analysis presented in Supplementary Figure S1, sTREM-1 concentrations are not different between moderate and severe illness in subgroups of survivors and non-survivors. It could be suggested that patients with more severe illness are at higher risk of death and therefore there is an overlap in the groups based on disease severity and mortality. When addressing this interaction, we observed that disease severity alone does not result in different sTREM-1 plasma concentrations whereas, sTREM-1 plasma concentrations are associated with mortality, independently of disease severity. Therefore, it can be concluded that the relationship of sTREM-1 concentrations and mortality is most robust.

Our results are in line with a recent study showing significantly higher sTREM-1 concentrations in COVID-19 patients that required intubation or died, compared to patients who were admitted to the hospital and survived without intubation [10]. Moreover, the authors report a good predictive accuracy of both sTREM-1 and IL-6 for disease severity and mortality. Similarly, we have observed that the prognostic value of sTREM-1 is better than that of other inflammatory markers such as CRP and ferritin, and similar to that of IL-6. sTREM-1 and IL-6 represent different aspects of the inflammatory response, but are both involved in the dysregulated inflammatory state seen in COVID-19 and sepsis patients. Therefore, we can speculate that a combination of these biomarkers could improve the identification of patients with an adverse outcome, which is also suggested by the results presented in Figure 4E. This validates the proposal that combinations of sTREM-1 with other clinical parameters and biomarkers might improve its prognostic potential, as shown previously in sepsis [9].

TREM-1 expression is upregulated following stimulation of human monocytes and neutrophils with various Toll-like receptor (TLR) ligands, including those implicated in antiviral response (mainly TLR3, 7, 8, 9). Co-activation of TREM-1 with these stimuli results in an increased production of cytokines [18–22]. Of note, coronaviruses are a large family of single-stranded RNA (ssRNA) viruses, ssRNA being mainly recognized by TLR7/8. Activated TREM-1 amplifies inflammatory responses in a pathogen agnostic manner. First of all, it promotes immune cell responses activated by the pattern recognition receptors (PRR). Moreover, TREM-1 activation was shown to result in a persistent release of cytokines and chemokines (tumour necrosis factor alfa [TNF-α], IL-1β, IL-8, and monocyte chemotactic protein [MCP]-1) [8], and is directly related to endothelial dysfunction [23] and platelet activation [24]. This might suggest a link between TREM-1 activation and the increased incidence of thromboembolic events seen in COVID-19, which is also supported by our data (Figure 2C). Because of the role of the TREM-1 pathway in the development of a dysregulated inflammatory response in some patients, it is thought that the pathway could contribute to poor outcomes in sepsis and COVID-19.

As studies in sepsis have indicated the TREM-1 pathway as a potential therapeutic target [11–13], it is tempting to speculate that, like anti-IL-6R therapy, a beneficial effect of TREM-1 modulation in patients with COVID-19 could be also present. Our data add to the hypothesis that anti-TREM-1 therapy could be effective in severely ill patients with COVID-19. This is further advocated by the analogous associations of sTREM-1 and IL-6 with mortality presented in the present study and the close relation of TREM-1 and IL-6 in the innate immune response to infections. A potential advantage of TREM-1 modulation is the targeting of the initial immune dysregulation as TREM-1 is an amplifier of the inflammatory response. Therefore, the immunosuppressive state reported in critically ill patients could potentially be avoided [13, 25].

The mechanisms described above, and the strong correlation of sTREM-1 with mortality, suggest that inhibition of TREM-1 may be beneficial in COVID-19. However, the timing of anti-inflammatory therapy in COVID-19 remains challenging. Early application might not be necessary or could diminish an effective immune response, whereas in advanced stages the hyperinflammation is already leading to adverse outcomes regardless of treatment [26]. As proposed earlier in the sepsis field, patient selection for host-directed therapies based on clinical biomarkers is crucial [27, 28]. sTREM-1 plasma concentrations might be useful for patient selection and follow-up of treatment effect and therefore serve as a prognostic or predictive biomarker for anti-TREM-1 therapy [13].

This study also has some limitations. First, the study populations are relatively small, and we tried to compensate for this by studying a discovery and validation cohort. Second, no longitudinal measurements of sTREM-1 plasma concentrations during hospital admission were performed, and a comprehensive longitudinal analysis of the TREM-1 pathway in disease deterioration was therefore not possible. Therefore, direct conclusions regarding causality cannot be drawn from our observational data and prospective studies are warranted. Third, a degree of variation in the timepoint of baseline sample collection was introduced because of the pragmatic design of the present study during the first wave of the pandemic; however, the differences in duration of symptoms or hospital stay at the time of sampling do not seem to have significantly influenced the results.

In conclusion, the present study shows that sTREM-1 plasma concentrations are increased in patients with COVID-19, and these concentrations are even higher in ICU patients and non-survivors. Moreover, discrimination between survivors and non-survivors based on sTREM-1 plasma concentration was possible. Therefore, it could be speculated that TREM-1 inhibition is a therapeutic target in COVID-19, and underlines the potential of a clinical trial of anti-TREM-1 treatment in patients with COVID-19, which is underway.

## Perspectives

- The TREM-1 pathway is known to amplify the inflammatory response in bacterial, viral, and fungal infections, resulting in the release of sTREM-1 into the blood. In patients with sepsis, increased sTREM-1 plasma concentrations are associated with mortality.
- sTREM-1 circulating concentrations are elevated in patients with COVID-19, especially in severely ill patients. Moreover, sTREM-1 is able to discriminate between survivors and non-survivors.
- Our data suggest that the TREM-1 pathway is involved in the hyperinflammatory response and is associated with adverse outcomes in COVID-19. Therefore, TREM-1 modulation might have clinical benefits in patients with COVID-19.

## Supplementary Material

Supplementary Figures S1-S3 and Tables S1-S2

## Data Availability

The datasets generated during and/or analysed during the present study are available from the corresponding author on reasonable request.

## Abbreviations

ARDS: acute respiratory distress syndrome
AUC: area under the curve
BMI: body mass index
COVID-19: coronavirus disease 2019
CRP: C-reactive protein
EDTA: ethylenediaminetetraacetic acid
ELISA: enzyme-linked immunosorbent assays
HC: healthy control
HR: hazard ratio
ICU: Intensive Care Unit
IL: interleukin
LOS: length of stay
MCP: monocyte chemotactic protein
PCR: polymerase chain reaction
PRR: pattern recognition receptors
ROC: receiver operating characteristic
SARS-CoV-2: severe acute respiratory syndrome coronavirus 2
ssRNA: single-stranded RNA
sTREM-1: soluble Triggering Receptor Expressed on Myeloid cells 1
TEE: thromboembolic event
TNF-α: tumour necrosis factor alfa
TLR: Toll-like receptor
TREM-1: Triggering Receptor Expressed on Myeloid cells 1
WBC: white blood cell

## References

[1] Guan W.J., Ni Z.Y., Hu Y., Liang W.H., Ou C.Q., He J.X. et al. (2020) Clinical Characteristics of Coronavirus Disease 2019 in China. N. Engl. J. Med. 382, 1708–1720 10.1056/NEJMoa2002032 32109013 PMC7092819

[2] Huang C., Wang Y., Li X., Ren L., Zhao J., Hu Y. et al. (2020) Clinical features of patients infected with 2019 novel coronavirus in Wuhan. China Lancet. 395, 497–506 10.1016/S0140-6736(20)30183-5 31986264 PMC7159299

[3] Zhou F., Yu T., Du R., Fan G., Liu Y., Liu Z. et al. (2020) Clinical course and risk factors for mortality of adult inpatients with COVID-19 in Wuhan, China: a retrospective cohort study. Lancet 395, 1054–1062 10.1016/S0140-6736(20)30566-3 32171076 PMC7270627

[4] Liu F., Li L., Xu M., Wu J., Luo D., Zhu Y. et al. (2020) Prognostic value of interleukin-6, C-reactive protein, and procalcitonin in patients with COVID-19. J. Clin. Virol. 127, 104370 10.1016/j.jcv.2020.104370 32344321 PMC7194648

[5] Herold T., Jurinovic V., Arnreich C., Lipworth B.J., Hellmuth J.C., von Bergwelt-Baildon M. et al. (2020) Elevated levels of IL-6 and CRP predict the need for mechanical ventilation in COVID-19. J. Allergy Clin. Immunol. 146, 128e4–136e4 10.1016/j.jaci.2020.05.008 32425269 PMC7233239

[6] Baindara P., Agrawal S. and Mandal S.M. (2020) Host-directed therapies: a potential solution to combat COVID-19. Expert Opin. Biol. Ther. 20, 1117–1120 10.1080/14712598.2020.1807001 32783643 PMC7441763

[7] Gordon D.E., Jang G.M., Bouhaddou M., Xu J., Obernier K., White K.M. et al. (2020) A SARS-CoV-2 protein interaction map reveals targets for drug repurposing. Nature 583, 459–468 10.1038/s41586-020-2286-9 32353859 PMC7431030

[8] Bouchon A., Facchetti F., Weigand M.A. and Colonna M. (2001) TREM-1 amplifies inflammation and is a crucial mediator of septic shock. Nature 410, 1103–1107 10.1038/35074114 11323674

[9] Su L., Liu D., Chai W., Liu D. and Long Y. (2016) Role of sTREM-1 in predicting mortality of infection: a systematic review and meta-analysis. BMJ Open 6, e010314 10.1136/bmjopen-2015-010314 27178971 PMC4874109

[10] Van Singer M., Brahier T., Ngai M., Wright J., Weckman A.M., Erice C. et al. (2021) COVID-19 risk stratification algorithms based on sTREM-1 and IL-6 in emergency department. J. Allergy Clin. Immunol. 147, 99e4–106e4 10.1016/j.jaci.2020.10.001 33045281 PMC7546666

[11] Wang F., Liu S., Wu S., Zhu Q., Ou G., Liu C. et al. (2012) Blocking TREM-1 signaling prolongs survival of mice with Pseudomonas aeruginosa induced sepsis. Cell. Immunol. 272, 251–258 10.1016/j.cellimm.2011.10.006 22055202

[12] Gibot S., Kolopp-Sarda M.N., Bene M.C., Bollaert P.E., Lozniewski A., Mory F. et al. (2004) A soluble form of the triggering receptor expressed on myeloid cells-1 modulates the inflammatory response in murine sepsis. J. Exp. Med. 200, 1419–1426 10.1084/jem.20040708 15557347 PMC2211948

[13] Francois B., Wittebole X., Ferrer R., Mira J.P., Dugernier T., Gibot S. et al. (2020) Nangibotide in patients with septic shock: a Phase 2a randomized controlled clinical trial. Intensive Care Med. 46, 1425–1437 10.1007/s00134-020-06109-z 32468087

[14] Prokop M., van Everdingen W., van Rees Vellinga T., Quarles van Ufford J., Stoger L., Beenen L. et al. (2020) CO-RADS - a categorical CT assessment scheme for patients with suspected COVID-19: definition and evaluation. Radiology 296, E97–E104 10.1148/radiol.2020201473 32339082 PMC7233402

[15] Zumla A., Hui D.S., Azhar E.I., Memish Z.A. and Maeurer M. (2020) Reducing mortality from 2019-nCoV: host-directed therapies should be an option. Lancet 395, e35–e36 10.1016/S0140-6736(20)30305-6 32035018 PMC7133595

[16] , Recovery Collaborative Group Horby P., Lim W.S., Emberson J.R., Mafham M., Bell J.L. et al. (2021) Dexamethasone in Hospitalized Patients with Covid-19. N. Engl. J. Med. 384, 693–704 10.1056/NEJMoa2021436 32678530 PMC7383595

[17] , REMAP-CAP Investigators Gordon A.C., Mouncey P.R., Al-Beidh F., Rowan K.M., Nichol A.D. et al. (2021) Interleukin-6 Receptor Antagonists in Critically Ill Patients with Covid-19. N. Engl. J. Med. 384, 1491–1502 10.1056/NEJMoa2100433 33631065 PMC7953461

[18] Zhu J., Duan G., Wang H., Cao M. and Liu Y. (2016) TREM-1 activation modulates dsRNA induced antiviral immunity with specific enhancement of MAPK signaling and the RLRs and TLRs on macrophages. Exp. Cell Res. 345, 70–81 10.1016/j.yexcr.2016.05.018 27237091

[19] Radsak M.P., Salih H.R., Rammensee H.G. and Schild H. (2004) Triggering receptor expressed on myeloid cells-1 in neutrophil inflammatory responses: differential regulation of activation and survival. J. Immunol. 172, 4956–4963 10.4049/jimmunol.172.8.4956 15067076

[20] Prufer S., Weber M., Sasca D., Teschner D., Wolfel C., Stein P. et al. (2014) Distinct signaling cascades of TREM-1, TLR and NLR in neutrophils and monocytic cells. J. Innate. Immun. 6, 339–352 10.1159/000355892 24281714 PMC6741579

[21] Netea M.G., Azam T., Ferwerda G., Girardin S.E., Kim S.H. and Dinarello C.A. (2006) Triggering receptor expressed on myeloid cells-1 (TREM-1) amplifies the signals induced by the NACHT-LRR (NLR) pattern recognition receptors. J. Leukoc. Biol. 80, 1454–1461 10.1189/jlb.1205758 16940328

[22] Bleharski J.R., Kiessler V., Buonsanti C., Sieling P.A., Stenger S., Colonna M. et al. (2003) A role for triggering receptor expressed on myeloid cells-1 in host defense during the early-induced and adaptive phases of the immune response. J. Immunol. 170, 3812–3818 10.4049/jimmunol.170.7.3812 12646648

[23] Jolly L., Carrasco K., Derive M., Lemarie J., Boufenzer A. and Gibot S. (2018) Targeted endothelial gene deletion of triggering receptor expressed on myeloid cells-1 protects mice during septic shock. Cardiovasc. Res. 114, 907–918 10.1093/cvr/cvy018 29361046

[24] Jolly L., Lemarie J., Carrasco K., Popovic B., Derive M., Boufenzer A. et al. (2017) Triggering receptor expressed on myeloid cells-1: a new player in platelet aggregation. Thromb. Haemost. 117, 1772–1781 10.1160/TH17-03-0156 28837205

[25] Boomer J.S., To K., Chang K.C., Takasu O., Osborne D.F., Walton A.H. et al. (2011) Immunosuppression in patients who die of sepsis and multiple organ failure. JAMA 306, 2594–2605 10.1001/jama.2011.1829 22187279 PMC3361243

[26] Siddiqi H.K. and Mehra M.R. (2020) COVID-19 illness in native and immunosuppressed states: a clinical-therapeutic staging proposal. J. Heart Lung Transplant. 39, 405–407 10.1016/j.healun.2020.03.012 32362390 PMC7118652

[27] van der Poll T., van de Veerdonk F.L., Scicluna B.P. and Netea M.G. (2017) The immunopathology of sepsis and potential therapeutic targets. Nat. Rev. Immunol. 17, 407–420 10.1038/nri.2017.36 28436424

[28] Stanski N.L. and Wong H.R. (2020) Prognostic and predictive enrichment in sepsis. Nat. Rev. Nephrol. 16, 20–31 10.1038/s41581-019-0199-3 31511662 PMC7097452

