# Supplementary material for: Increased sTREM-1 plasma concentrations are associated with poor clinical outcomes in patients with COVID-19
Source: Biosci Rep. 2021 Jul 22;41(7):BSR20210940. doi: 10.1042/BSR20210940 (PMC8298260; doi:10.1042/BSR20210940)
Supplement: Supplementary Figures S1-S3 and Tables S1-S2 [file BSR-2021-0940_supp.pdf]

Supplementary Figure legends

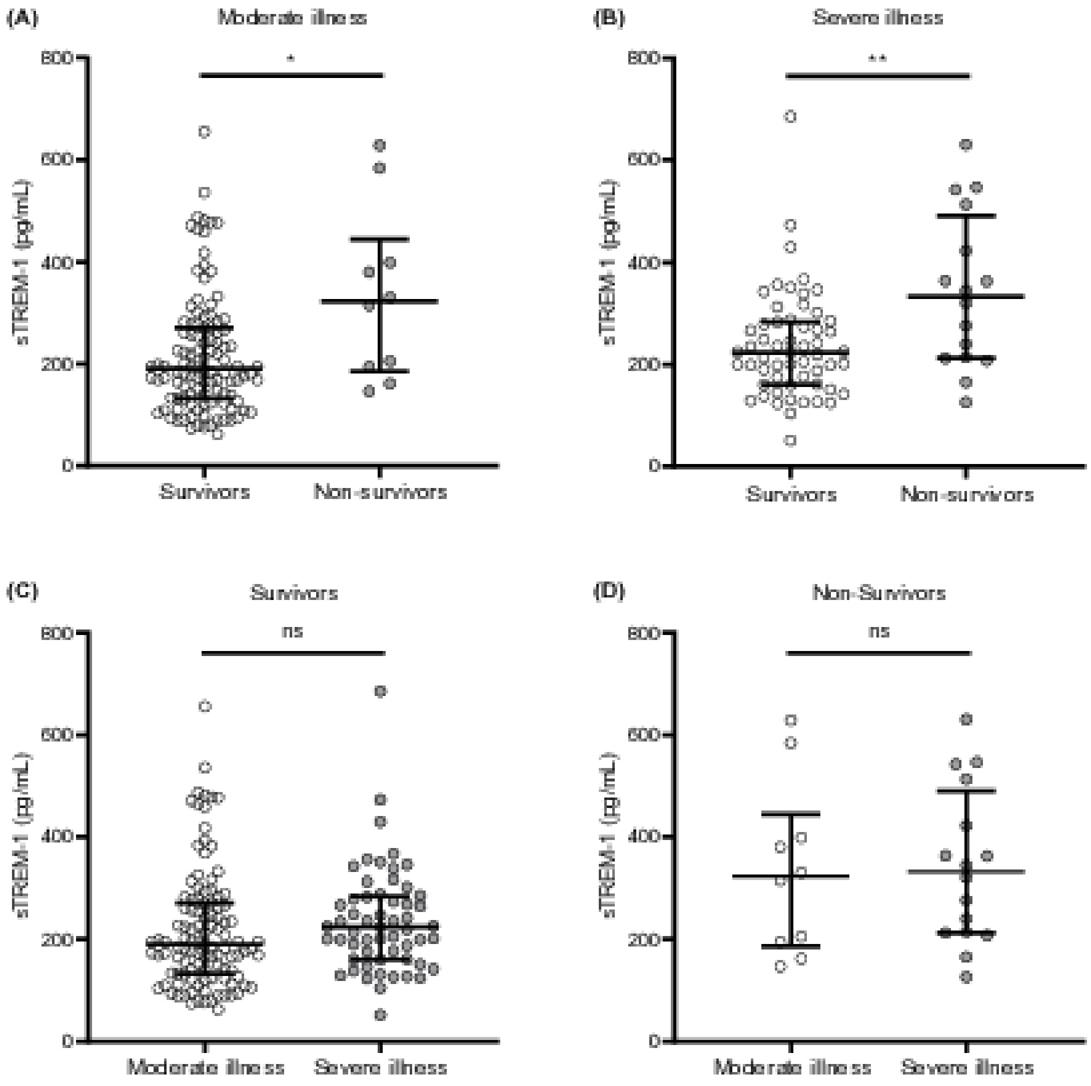

Supplementary Figure 1

sTREM-1 plasma concentrations in different groups of patients with COVID-19

sTREM-1 plasma concentration for (**A**) moderately ill patients with COVID-19 divided into survivors and non-survivors, (**B**) severely patients with COVID-19 divided into survivors and non-survivors, (**C**) surviving patients with COVID-19 divided into moderate and severe illness, and (**D**) non-surviving patients with COVID-19 divided into moderate and severe illness. Severe illness was defined as the need for ICU admission during hospital stay. Data are presented as median with interquartile range. Exact *P*-values are (**A**) 0.019, (**B**) 0.007, (**C**) 0.068, (**D**) 0.737. P-values were calculated with Mann-Whitney U tests. *: $P<0.05$, **: $P<0.01$. Abbreviations: COVID-19, coronavirus disease 2019; sTREM-1, soluble Triggering Receptor Expressed on Myeloid cells 1; ICU, intensive care unit; ns, not significant.

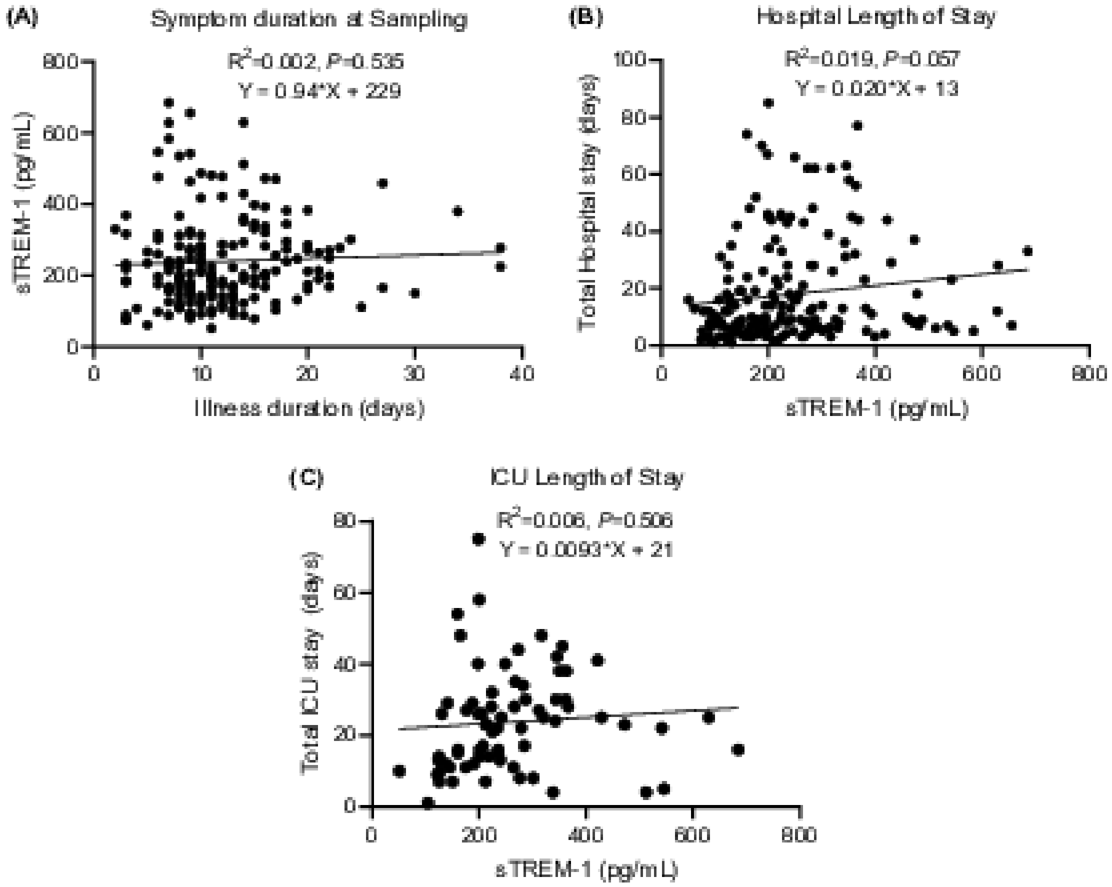
(A)
Symptom duration at Sampling
R2=0.002, P=0.535
Y = 0.94*X + 229
sTREM-1 (pg/mL)
Illness duration (days)
(B)
Hospital Length of Stay
R2=0.019, P=0.057
Y = 0.020*X + 13
Total Hospital stay (days)
sTREM-1 (pg/mL)
(C)
ICU Length of Stay
R2=0.006, P=0.506
Y = 0.0093*X + 21
Total ICU stay (days)
sTREM-1 (pg/mL)

Supplementary Figure 2

Associations of sTREM-1 and other clinical outcomes in patients with COVID-19

Simple linear regression assessing the influence of (**A**) symptom duration at time of sampling on sTREM-1 concentrations, (**B**) sTREM-1 concentrations on total length of hospital stay, and (**C**) sTREM-1 concentrations on total length of ICU stay. Abbreviations: COVID-19, coronavirus disease 2019; sTREM-1, soluble Triggering Receptor Expressed on Myeloid cells 1; ICU, intensive care unit.

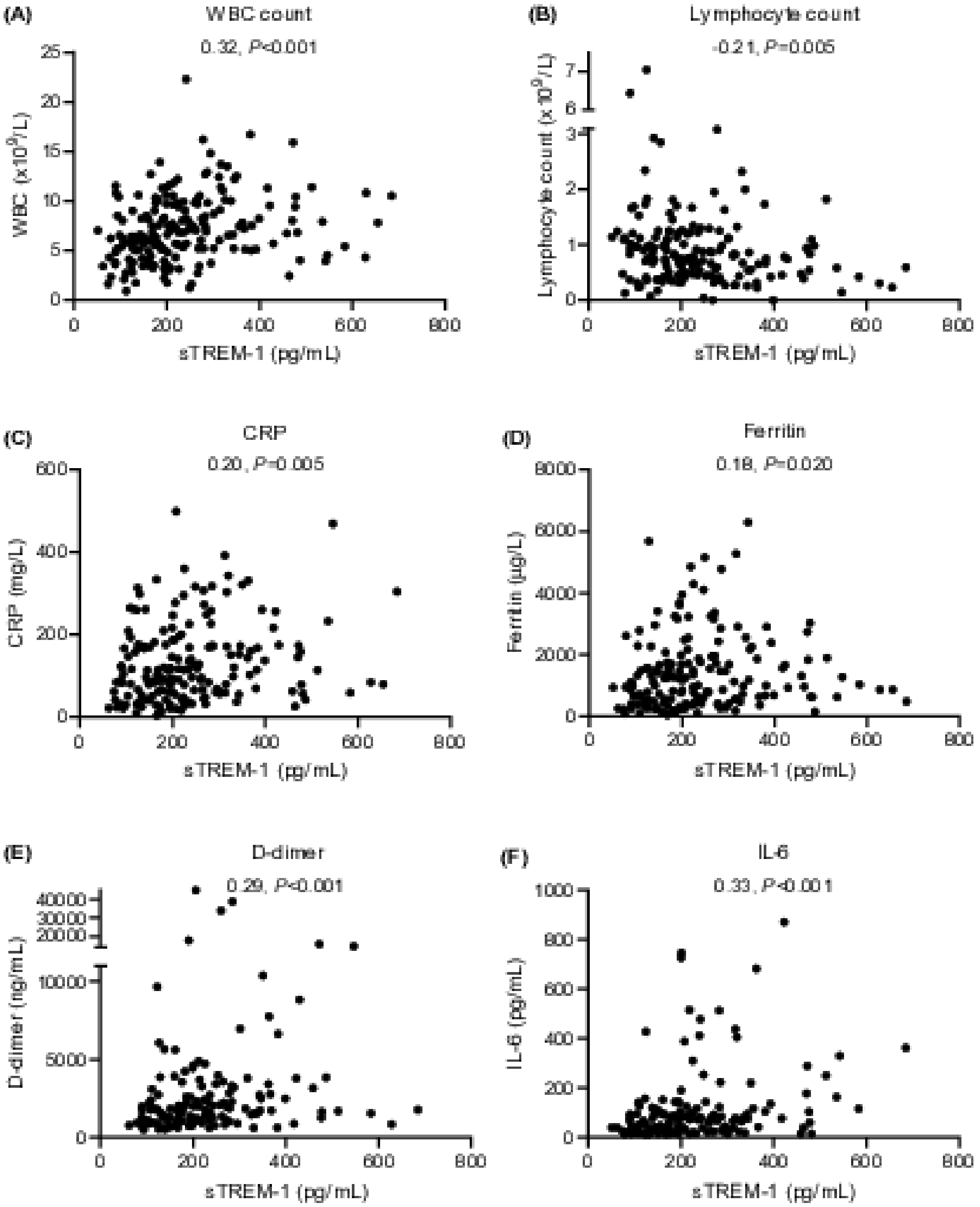
(A)
WBC count
0.32, P<0.001
WBC (x10^9/L)
sTREM-1 (pg/mL)
(B)
Lymphocyte count
-0.21, P=0.005
Lymphocyte count (x 10^9/L)
sTREM-1 (pg/mL)
(C)
CRP
0.20, P=0.005
CRP (mg/L)
sTREM-1 (pg/mL)
(D)
Ferritin
0.18, P=0.020
Ferritin (µg/L)
sTREM-1 (pg/mL)
(E)
D-dimer
0.29, P<0.001
D-dimer (ng/mL)
sTREM-1 (pg/mL)
(F)
IL-6
0.33, P<0.001
IL-6 (pg/mL)
sTREM-1 (pg/mL)

Supplementary Figure 3

Correlations of sTREM-1 and inflammatory markers in patients with COVID-19

Correlations of sTREM-1 and (**A**) white blood cell count, (**B**) lymphocyte count, (**C**) CRP, (**D**) ferritin, (**E**) D-dimer, and (**F**) IL-6 circulating concentrations. Correlation coefficients and *P*-values were calculated using Spearman's rank correlation test. Spearman's rho and *P*-values are shown in the figure. Abbreviations: COVID-19, coronavirus disease 2019; sTREM-1, soluble Triggering Receptor Expressed on Myeloid cells 1; WBC, white blood cell; CRP, C-reactive protein; IL-6, interleukin-6.

Supplementary Table S1. Summary of the analytical performances and acceptance criteria of the sTREM-1 ELISA validated method.

**Analyte**	sTREM-1	
**Matrix**	Human K_2-EDTA plasma	
**Analytical method**	ELISA	
**Calibration standards (two wells per CS including anchoring points#) in Assay Diluent from the kit**	15.6#, 31.3 (LCS), 62.5, 125, 250, 500, 750, 1000, 2000 (HCS) and 3000# pg/mL<br>The imprecision (%CV), must be ≤20% for CS ranged from 31.3 (LCS) to 2000 pg/mL (HCS).<br>The inaccuracy(%RE), must be within ±20% (±25% at the LCS and the HCS).<br>No more than two CS (30%) may be excluded from the calibration curve, which must contain finally at least 6 calibration concentration levels, including the LCS and HCS.	
**LLOQ in human K_2-EDTA plasma**	34.2 pg/mL	
**ULOQ in human K_2-EDTA plasma**	2070 pg/mL	
**QC samples**<br>**(n=2 duplicates, unspiked or spiked samples prepared in undiluted or diluted human K_2-EDTA plasma)**	QC.Low (93 pg/mL), QC.Mid (728 pg/mL) and QC.High (1610 pg/mL)<br>%CV must be ≤20% and %RE must be within ±20%.<br>At least 4/6 QC samples must be within acceptance criteria (2/6 QC samples, not both at the same concentration, may be outside the acceptance criteria).	
**MRD**	None	
**Dilution linearity**	Up to 1/100 in calibrator diluent RD5-18	
**Specificity against endogenous matrix components**	No interference was demonstrated in any of the blank human K_2-EDTA plasma samples tested.	
**Stability in human K_2-EDTA plasma**	Freeze/thaw (F/T) stability	3 cycles at -24°C±6°C<br>3 cycles at -75°C±10°C
	Short-term stability	24 hours at room temperature<br>24 hours at +5°C±5°C
	Long-term (LT) stability	670 days at -24°C±6°C<br>670 days at -75°C±10°C

LCS: lowest calibration standard; HCS: highest calibration standard; CS: calibration standard(s); QC: quality control; RE: relative error; MRD: minimum required dilution; LLOQ: lower limit of quantification; ULOQ: upper limit of quantification

Supplementary Table S2. Patient characteristics pilot cohort

	Healthy controls (n=21)	COVID-19 (n=24)
Age (years)	42 (22-48.5)	63 (58-71)
Gender (n, %)		
Male	9 (43)	18 (75)
Female	12 (57)	6 (25)
BMI (kg/m²)	NA	30.3 (27.4-33.8)
Comorbidity (n, %)		
Diabetes mellitus	NA	4 (17)
Cardiovascular disease		1 (4)
Pulmonary disease		1 (4)
Malignancy		0 (0)
Length of hospital stay (days)	NA	17 (10-20)
MV duration (days)	NA	15 (8-17)
Mortality (n,%)	NA	3 (13)
sTREM-1 (pg/mL)	104 (75-124)	161 (129-196)
IL-6 (pg/mL)	NA	172 (95-232)

Data are presented as median (IQR) or n (%). Abbreviations: COVID-19, coronavirus disease 2019; ICU, intensive care unit; BMI, body mass index; MV, mechanical ventilation, sTREM-1, soluble Triggering receptor Expressed on Myeloid cells 1; IL-6, interleukin-6.
